# Characterisation of the Xenogeneic Immune Response to Microencapsulated Fetal Pig Islet-Like Cell Clusters Transplanted into Immunocompetent C57BL/6 Mice

**DOI:** 10.1371/journal.pone.0059120

**Published:** 2013-03-15

**Authors:** Vijayaganapathy Vaithilingam, Cherry Fung, Sabina Ratnapala, Jayne Foster, Vijesh Vaghjiani, Ursula Manuelpillai, Bernard E. Tuch

**Affiliations:** 1 Diabetes Transplant Unit, Prince of Wales Hospital, School of Medical Sciences, University of New South Wales, Randwick, New South Wales, Australia; 2 Centre for Reproduction and Development, Monash Institute of Medical Research, Monash University, Clayton, Victoria, Australia; 3 Materials, Science and Engineering, Commonwealth Scientific and Industrial Research Organization, North Ryde, New South Wales, Australia; University of Bremen, Germany

## Abstract

Xenotransplantation of microencapsulated fetal pig islet-like cell clusters (FP ICCs) offers a potential cellular therapy for type 1 diabetes. Although microcapsules prevent direct contact of the host immune system with the xenografted tissue, poor graft survival is still an issue. This study aimed to characterise the nature of the host immune cells present on the engrafted microcapsules and effects on encapsulated FP ICCs that were transplanted into immunocompetent mice. Encapsulated FP ICCs were transplanted into the peritoneal cavity of C57BL/6 mice. Grafts retrieved at days 1, 3, 7, 14 and 21 post-transplantation were analysed for pericapsular fibrotic overgrowth (PFO), cell viability, intragraft porcine gene expression, macrophages, myofibroblasts and intraperitoneal murine cytokines. Graft function was assessed *ex vivo* by insulin secretion studies. Xenogeneic immune response to encapsulated FP ICCs was associated with enhanced intragraft mRNA expression of porcine antigens MIP-1α, IL-8, HMGB1 and HSP90 seen within the first two weeks post-transplantation. This was associated with the recruitment of host macrophages, infiltration of myofibroblasts and collagen deposition leading to PFO which was evident from day 7 post-transplantation. This was accompanied by a decrease in cell viability and loss of FP ICC architecture. The only pro-inflammatory cytokine detected in the murine peritoneal flushing was TNF-α with levels peaking at day 7 post transplantation. This correlated with the onset of PFO at day 7 implying activated macrophages as its source. The anti-inflammatory cytokines detected were IL-5 and IL-4 with levels peaking at days 1 and 7, respectively. Porcine C-peptide was undetectable at all time points post-transplantation. PFO was absent and murine intraperitoneal cytokines were undetectable when empty microcapsules were transplanted. In conclusion, this study demonstrated that the macrophages are direct effectors of the xenogeneic immune response to encapsulated FP ICCs leading to PFO mediated by a combination of both pro- and anti-inflammatory cytokines.

## Introduction

Porcine islet xenotransplantation offers a potential treatment for type 1 diabetes and a partial solution to the problem of human donor tissue shortage. Sources of porcine tissue include adult porcine islets, neonatal porcine pancreatic cell clusters (NPCCs) and fetal pig islet-like cell clusters (FP ICCs). Although adult porcine islets and NPCCs have been extensively studied in varied transplantation settings [1]–[3], FP ICCs have received less attention. As with NPCCs, FP ICCs are easily isolated [4], and are relatively resistant to hypoxia and attack by the pro-inflammatory cytokines that destroy adult β cells [5], [6]. A disadvantage of using FP ICCs is the low proportion of mature insulin-producing β cells at the time of transplantation compared to adult pig islets. However, as with NPCCs, following transplantation, most of the primitive duct cells differentiate into insulin-producing β cells [4]. Preclinical studies from our group have shown that FP ICCs can normalize blood glucose levels (BGLs) of diabetic recipients both when transplanted beneath the renal capsule of immunodeficient mice [7] and when allografted into an immunosuppressed pig [8]. Human trials with FP ICCs implanted into immunosuppressed diabetic recipients conferred neither a confirmed functional benefit nor any adverse consequences with some cells staining positive for insulin [9], [10]. One of the major problems facing the use of porcine islet tissue is the xenograft rejection by the host immune system and the need for chronic immunosuppression to prolong graft survival.

Encapsulation of porcine tissue using alginate hydrogels has been widely used to overcome immune rejection and prevent the need for anti-rejection drugs [11]. Previous studies from our group and others have shown that encapsulation of FP ICCs or NPCCs in alginate microcapsules enhanced graft survival and normalised BGLs when transplanted into the peritoneal cavity of SCID or nude diabetic mice [12], [13]. Despite these promising results in immunodeficient animals, data from immunocompetent animals have been inconsistent. Some investigators have shown that encapsulated porcine islets contained within barium-alginate or calcium-alginate microcapsules functioned in mice and non-human primates [14]–[16], whereas others have observed poor graft survival [17], [18]. Further, a human trial with microencapsulated NPCCs did not offer a major clinical benefit with only a slight reduction in the recipient's daily insulin requirements despite the microcapsules remaining intact for at least 9.5 years post-transplantation and there being a small number of glucose-responsive islets at this time [19]. Although microencapsulation prevents direct interaction of the graft and the host immune system, leakage of xenoantigens from the encapsulated cells may provoke an inflammatory and/or immune response [17]. A greater understanding of the host immune response to xenografted encapsulated FP ICCs is required before it might be considered for application clinically. This study aimed to examine the xenogeneic immune response to microencapsulated FP ICCs and effect on graft survival and function when transplanted into the peritoneal cavity of immunocompetent C57BL/6 mice. Our results suggest macrophages and myofibroblasts as direct effectors of the xenogeneic immune response to encapsulated FP ICCs leading to PFO and graft failure which is mediated by a combination of both pro- and anti-inflammatory cytokines.

## Materials and Methods

### Source of FP ICCs and mice

A total of 18 large White Landrace fetal pigs with an average gestational age of 78 days as determined by the crown rump length obtained from QAF Meats (Corowa, NSW, Australia) were used as the source of ICCs. Immunocompetent, normoglycemic male C57BL/6 mice (n = 20) were used as the transplantation recipients. All animal work was approved by the Animal Care and Ethics Committee of the University of New South Wales.

### Isolation of FP ICCs

Pancreata were removed from the fetal pigs under sterile conditions and placed in a 30 mm petri dish containing phosphate-buffered saline (PBS). Briefly, 1 g of pancreatic tissue was finely minced and then enzymatically digested using Collagenase P (3 mg/ml; Boehringer-Mannheim, Mannheim, Germany) following a previously published protocol [12]. Isolated cells were then cultured in modified RPMI-1640 medium (Gibco, Mulgrave, VIC, Australia) supplemented with 10% fetal calf serum (FCS) (Sigma, St Louis, MO) at 37°C in 5% CO_2_ for 3 days to allow the cells to round up and form ICCs.

### Encapsulation

On day three post-isolation, ICCs were washed with 0.9% NaCl and centrifuged at 40× *g* for 5 min. After removal of supernatant, the ICCs were suspended in highly purified 2.2% alginate (60∶40 guluronic acid: mannuronic acid; UP MVG Pronova, FMC Biopolymer, Sandvika, Norway) solution in a 1∶6 ratio. The microcapsule formation was carried out in an air-driven droplet generator (Steinau, Berlin, Germany) as described previously [12] with an air flow rate of 8 l/min at a pressure of 100 kPa. The barium alginate microcapsules were then washed thrice in 0.9% saline with subsequent removal of the supernatant in between each washing to sieve off the smaller microcapsules which take a longer time to settle. The average diameter of the barium alginate microcapsules was found to be 244.9±13.9 µm (Figure S1). Encapsulated FP ICCs were then cultured overnight in a modified RPMI-1640 medium (Gibco) at 37°C in a humidified atmosphere with 5% CO_2_ and air.

### Viability

The viability of encapsulated FP ICCs was assessed using the fluorescent dyes 6-carboxyfluorescein diacetate (6-CFDA; Sigma) and propidium iodide (PI; Sigma). The percentage of cells with green fluorescence (live) to red (dead cells) was assessed to evaluate viability (n = 100 capsules, for each time point and preparation). Samples were analysed under a fluorescent microscope (Axioskop 2, Zeiss, Berlin, Germany and using Axiovision LE software).

### Insulin secretion and content

To measure the insulin content and secretion ability of FP ICCs, static stimulation in response to a stimulus *in vitro* was performed. Unlike adult β cells, fetal β cells are immature and do not secrete insulin in response to glucose. Instead, potassium chloride (KCl) was used as the stimulus since it has the ability to depolarise cell membranes of both immature and mature β cells, leading to an influx of calcium and stimulate insulin secretion [20]. Aliquots of encapsulated FP ICCs were randomly hand picked and washed with HEPES-buffered Earles Medium (Gibco) containing 0.2% bovine serum albumin (Sigma) (HBEM-BSA). Approximately 100 encapsulated FP ICCs were either exposed to basal HBEM-BSA medium or stimulatory medium containing 20 mM KCl in HBEM-BSA (n = 3; for both basal and stimulus and each containing 100 encapsulated FP ICCs) for 1 h at 37°C with gentle agitation. After 1 h, the supernatant was collected and stored at –80°C for the measurement of secreted insulin by radioimmunoassay (RIA) (Linco, St. Charles, MI). The cell pellet was washed in Hanks Balanced Salt Solution (HBSS; Sigma) followed by the addition of cold acid ethanol and vortexed vigorously to enhance cell lysis. The cell extract was kept at 4°C overnight and supernatant collected the following day and stored at −20°C for measurement of insulin content by RIA.

### Transplantation of microencapsulated FP ICCs

Approximately 8000 microencapsulated FP ICCs with a graft volume of 400 µl were transplanted into the peritoneal cavity of each immunocompetent normoglycemic C57BL/6 mouse (n = 20). Briefly, the mice were anaesthetised with pentobarbitone (70 mg/kg) and the encapsulated FP ICCs were infused into the peritoneal cavity using a 14-gauge catheter *via* a 3 ml syringe. The catheter was flushed twice with 1 ml of 0.9% sterile saline to transfer all remaining microcapsules, then removed and the peritoneum closed by a purse-string suture and the skin stapled. Controls consisted of mice (n = 4) transplanted with an equivalent number of empty microcapsules (400 µl) into the peritoneal cavity.

### Graft retrieval and collection of peritoneal fluid

At days 1, 3, 7, 14 and 21 post-transplantation, four recipient mice were killed at each of these time-points. Beating heart blood was collected from recipient mice, centrifuged at 15,000× *g* for 5 min and serum stored at −80°C until analysis by RIA for porcine C-peptide. Capsules were retrieved by flushing the peritoneal cavity with 5 ml HBSS containing 0.06% BSA, 1% penicillin and streptomycin, 2 mM L-glutamine and 10 mM HEPES (Sigma), at pH 7.4. The microcapsules were removed from the peritoneal lavage fluid by gravity separation. The resulting peritoneal fluid was then spun at 3000 rpm to remove immune cells and debris, and then frozen at −80°C for future analysis of chemokines/cytokines. The peritoneal cavity was flushed with 0.9% NaCl and subsequently examined for any remaining capsules. The packed cell volume of the graft recovered was recorded. Empty capsules were retrieved from control mice (n = 4) at day 21 post-transplantation. A random aliquot of microcapsules were then taken for morphological assessment, viability, static stimulation with KCl, real-time PCR and histological analysis.

### Morphological assessment of retrieved graft

Retrieved capsules (n = 100) from each recipient mouse were examined under a light microscope for the degree of cellular/fibrotic overgrowth using a scoring system as described previously [21] where score 0  =  no overgrowth, score 1 = <25% of overgrowth, score 2 = 25–50%, score 3 = 51–75% and score 4 = >75% of overgrowth.

### Decapsulation and RNA extraction

Aliquots of encapsulated ICCs were decapsulated using a solution containing 0.5 M EDTA (Gibco) and 1 M HEPES. RNA extraction was undertaken using the RNeasy Mini Kit (Qiagen, Valencia, CA), following manufacturer's protocols. Following RNA integrity testing, the RNA was converted to cDNA using the Superscript First-Strand synthesis system (Invitrogen) in a Myocycler ® thermal cycler (Bio-Rad, Hercules, CA) and stored at −20°C for later quantitative real-time RT-PCR analysis.

### Real-time PCR

cDNA samples from ICCs retrieved at 3, 7, 14 and 21 days after transplantation were analysed by quantitative real-time PCR using TaqMan fluorescent (FAM)-labelled probes and primers (Sigma-Genosys, Sydney, Australia). The probe/primer pair sequences are shown in Table S1. All primers used were desalted and all probes were purified by HPLC. The probe/primer sets were designed using Primer Express (Applied Biosystems, Carlsbad, CA). The PCR reactions contained 0.25 µl of 10 µM forward and reverse primers, 0.25 µl of 10 µM appropriate TaqMan probe, 12.5 µl of 2× universal buffers, and 5 µl of 0.2 µg/µl cDNA in a total reaction volume of 25 µl. To verify the quality of the cDNA, samples from each time point were run with porcine glyceraldehyde-3 phosphate dehydrogenase (GAPDH) primers. Real time analysis was done using the ABI Prism 7700 Sequence Detection System® (Applied Biosystems). The PCR conditions were as follows: 50 °C for 2 min, 95°C for 10 min, 95°C for 15 sec, then 60°C for 1 min. Expression of target genes was normalized to GAPDH and expressed as a fold change.

### Histological analyses

#### Haematoxylin & Eosin staining

Microencapsulated FP ICCs were washed twice in PBS and incubated in 10% neutral buffered formalin overnight. Capsules were washed again in PBS and suspended in 2% agarose and embedded in paraffin. These were cut into 5 µm sections using (820 Spencer Microtome, Labequip, Ontario, Canada). Sections were stained with haematoxylin and eosin (H&E) using a Shandon Varistain 24-4 staining machine® (Thermo Scientific, Kalamazoo, MI) to examine the morphology of FP ICCs.

#### Insulin staining

Paraffin embedded capsules, prepared as for H&E staining, were cut into 5 µm thick sections and stained for insulin. Sections were deparaffinised and rehydrated followed by guinea pig anti-insulin antibody (1∶500; Dako, Glostrup, Denmark). The secondary antibody rabbit anti-guinea pig tetramethyl rhodamine isothiocyanate (TRITC) conjugated immunoglobulins (1∶1000 dilution; Sigma) was used to detect the bound primary antibody. Cell nuclei were stained with 4,6-diamidino-2-phenylindole (DAPI) (1∶10000 dilution; Molecular Probes, Carlsbad, CA). Sections were viewed under a fluorescent microscope (Axioskop 2, Zeiss using Axiovision LE software).

#### Collagen staining

Paraffin embedded capsules, prepared as for H&E staining, were cut into 5 µm thick sections and stained for collagen. Briefly, the sections were deparaffinised and rehydrated followed by nuclear staining with haematoxylin. The sections were then washed for 10 min in running tap water and stained with picro-sirius red for 1 h. After two washes in acidified water, sections were dehydrated in three changes of 100% ethanol followed by a final wash in xylene. The sections were then mounted and viewed under a bright field microscope (Olympus IX81, Center Valley, PA).

#### Immunostaining for macrophages and myofibroblasts

Paraffin embedded capsules were cut into 5 µm thick sections. The sections were dewaxed and rehydrated. Macrophages were detected with rat anti-mouse F4/80 (1∶4000; Abcam; Cambridge, UK) and myofibroblasts with mouse anti-human α-smooth muscle actin (α-SMA) antibody which cross reacts with mouse (1∶5000; Sigma-Aldrich). Endogenous peroxidase was quenched with 0.6% H_2_O_2_ and non specific binding blocked with CAS block (Invitrogen; Camarillo, CA). Primary antibodies were incubated at 4 °C overnight (F4/80) or 30 min at room temperature (α-SMA). Primary antibodies were omitted from negative controls. After three washes, sections were incubated with biotinylated goat anti-mouse IgG (Vector Laboratories, Burlingame, CA; 1∶200; α-SMA) or biotinylated rabbit anti-rat IgG (Dako; Glostrup, Denmark; 1∶150; F4/80). Antibody binding was detected using the ABC kit (Vector Laboratories) followed by DAB (Sigma-Aldrich) as chromagen. Slides were counter stained with haematoxylin, dehydrated and mounted with DPX (Sigma-Aldrich).

### Porcine C-peptide assay

Frozen samples of murine serum were thawed and levels of C-peptide were quantified using a commercial porcine C-peptide RIA kit (Linco) according to supplier's protocol. Centrifugation was performed at 4200×*g* for 40 min to prevent pellet loss while decanting the supernatant. Serum from mice that did not undergo transplantation was used as the negative control.

### Quantification of mouse cytokines

Frozen samples (1.5 ml) of mouse peritoneal fluid were thawed and concentrated at 250×*g* for 1.5 h at 40°C using a MiVac Duo Concentrator centrifuge (Genevac, Gardiner, NY). This was followed by further centrifugation at 250×*g* for 1 h at 45°C to further concentrate the samples to 100 µl (15× concentrated). Mice transplanted with empty capsules served as controls. Concentrated samples were analysed using a commercial multiplex bead-based immunoassay kit for mouse IL-1β, IL-2, IL-4, IL-5, IL-10, IL-12, TNF-α and IFN-γ (Linco), according to supplier's protocols. HBSS was used as the matrix solution. Mouse peritoneal cytokines were then quantified using a Bio-plex (Bio-Rad) suspension array system. Data were analysed from the standard curves generated by Bio-plex manager v4.1.1 (Bio-Rad).

### Statistical analysis

All data are presented as mean ± standard error of mean (SEM). Differences between two groups were analysed by the two-tailed Student's t-test and of more than two groups by one-way ANOVA with *post-hoc* Tukey-Kramer Multiple-Comparison test. The software NCSS 2004 (NCSS, Kaysville, UT) was used to perform the statistical data analysis. Significant differences among data groups were assigned when *p*<0.05.

## Results

### Graft retrieval

#### Graft recovery decreased with time post-transplantation

**Figure 1:**
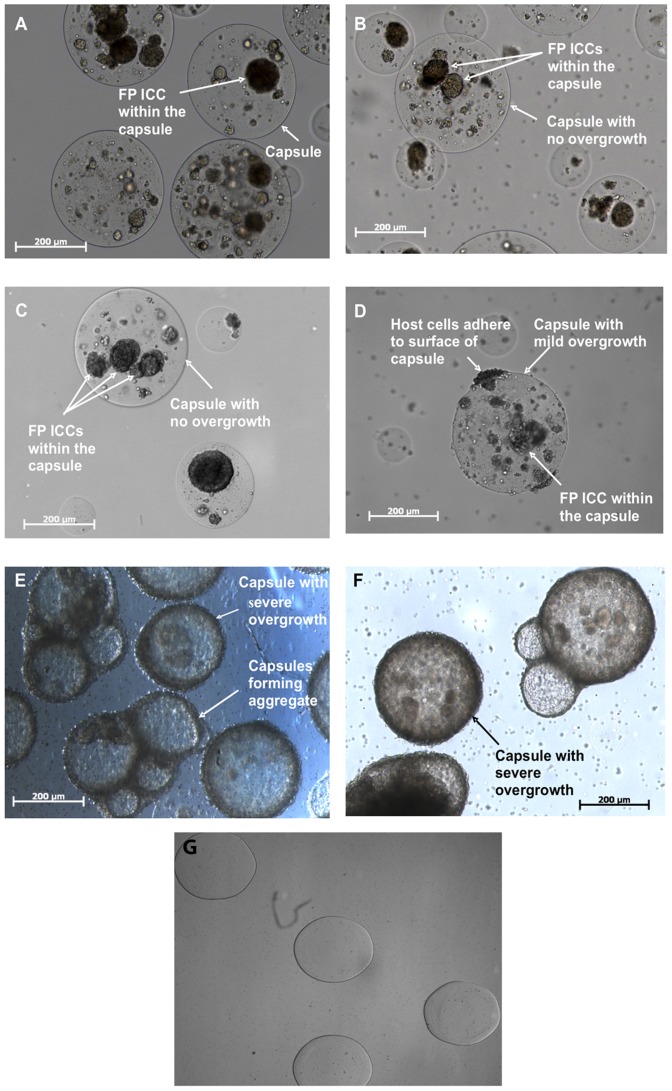
Light micrographs of microencapsulated FP ICCs. (A) Microencapsulated FP ICCs pre-transplantation, (B) day 1 post-transplantation, (C) day 3 post-transplantation, (D) day 7 post-transplantation, (E) day 14 post-transplantation and (F) day 21 post-transplantation. No capsule overgrowth was observed at days 1 and 3 post-transplantation (panels B and C). Capsule overgrowth was evident at days 7 to 21 post-transplantation (panels D to F), with an example of mild overgrowth (score  = 1) shown in panel (D), and examples of severe overgrowth (score  = 4) shown in panels (E and F). (G) Empty microcapsules explanted at day 21 post-transplantation had no fibrotic overgrowth (score  = 0).

Empty microcapsules retrieved at day 21 post-transplantation remained round and intact with no fibrosis or capsule breakage (Figure 1G). This indicates that the barium-alginate microcapsules were biocompatible and were not degraded in the recipient. With morphologies similar to the pre-transplantation controls, no pericapsular fibrotic overgrowth (PFO) was observed on the surface of capsules containing FP ICC at days 1 and 3 post-transplantation (Figures 1A–C; Table 1) However, at day 7 post-transplantation, PFO was observed, with scores ranging from 0 to 4. About half of the capsules had either no or only mild overgrowth (Figure 1D). At days 14 and 21 post-transplantation, over 80% of the capsules examined had an overgrowth score of 4, indicating severe PFO. The majority of the capsules had host cells covering the whole surface of the capsule and some of the capsules had formed aggregates (Figures 1E&F). The PFO at days 7 to 21 post-transplantation correlated with the decrease in graft recovery at these times. Only free floating capsules were recovered from the flushing of the peritoneal cavity by modified HBSS. Compared to days 1 and 3, at day 7 post-transplantation, the graft recovery rate was only 66±13% and this decreased further to 56±6% by day 21 (Table 2). The decrease in graft recovery may have been due to the attachment of the capsules with PFO to abdominal organs. The graft recovery rate was 100% for empty microcapsules retrieved at day 21 post-transplantation due to the absence of PFO (Figure 1G).

**Table 1:** Assessment of the degree of PFO.

Time post-transplantation	Median overgrowth score*	Capsule overgrowth score (% of capsules examined)				
		0	1	2	3	4
Day 1	0	100±0	0±0	0±0	0±0	0±0
Day 3	0	100±0	0±0	0±0	0±0	0±0
Day 7	1	34±12	17±4	22±5	16±1	11±2
Day 14	4	10±4	4±1	1±0	2±1	84±4
Day 21	4	6±2	1±1	2±1	0±0	90±3

* 0 = no overgrowth, 1 = <25% of overgrowth, 2 = 25–50% of overgrowth, 3 = 51–75% of overgrowth and 4 = >75% of overgrowth. Values are mean ± SEM (n = 4 mice for each time point).

**Table 2:** Graft recovery rates.

Time post-transplantation	Volume of graft recovered (µl)	Graft recovery (%)
Day 1	380±10	94±3
Day 3	390±5	96±1
Day 7	260±50	66±13
Day 14	240±24	59±6
Day 21	230±25	56±6

The grafts were retrieved at different time points post-transplantation and the recovered graft volume recorded. Values are mean ± SEM (n = 4 mice for each time point).

#### Graft viability decreased with time post-transplantation

**Figure 2:**
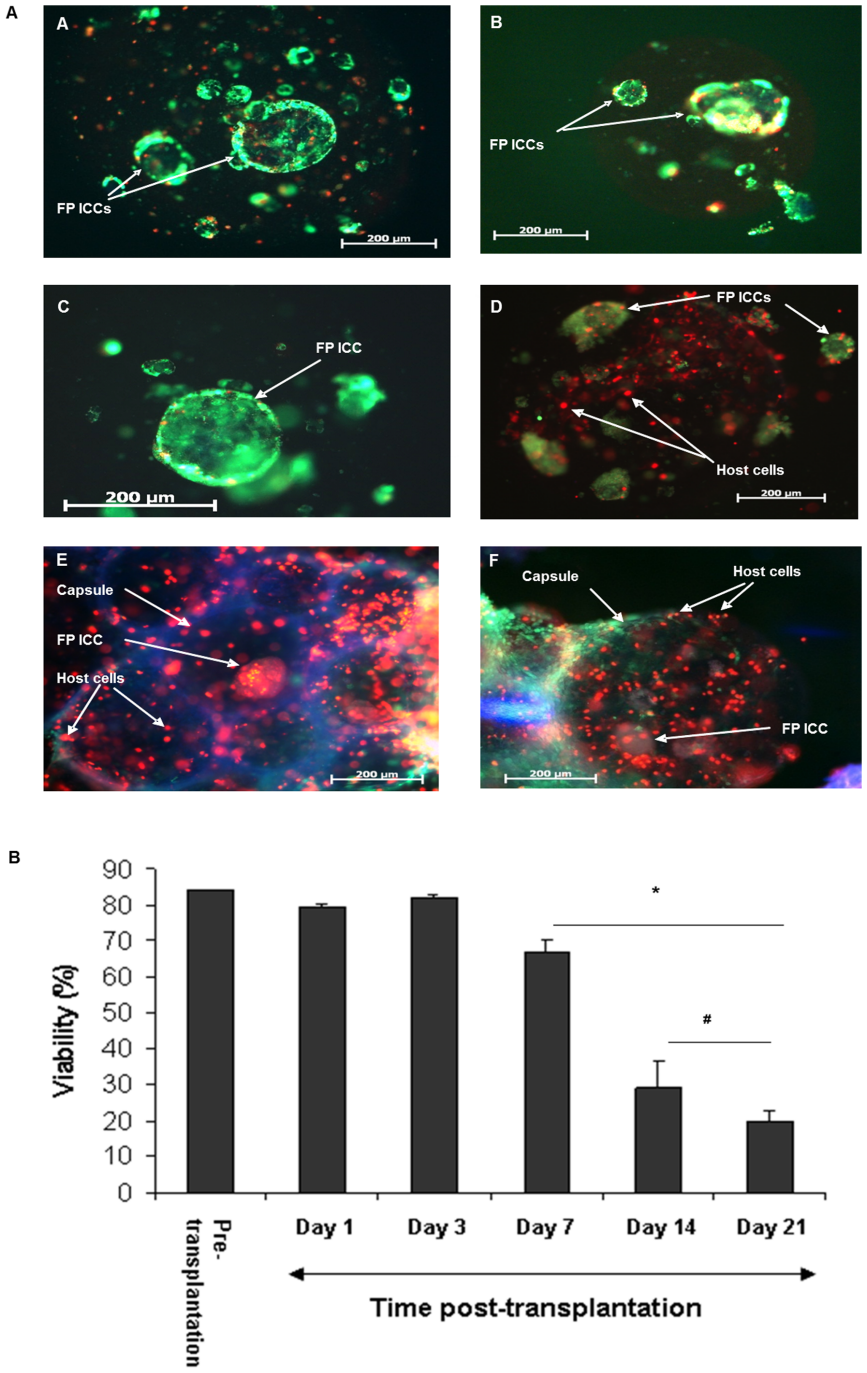
Viability of microencapsulated FP ICCs decreased with time post-transplantation. A) Fluorescent images of encapsulated FP ICCs stained with 6-CFDA for viable cells (green) and PI for non-viable cells (red) to assess viability at (A) pre-transplantation, (B) day 1, (C) day 3, (D) day 7, (E) day 14 and (F) day 21 post-transplantation. B) Viability decreased with time post-transplantation. Values are mean ± SEM (n = 100 microcapsules/mice for each time point); *p<0.05 viability for days 7 to 21 when compared to pre-transplantation control and days 1 and 3 post-transplantation; #p<0.05 viability for days 14 and 21 when compared to day 7 post-transplantation (one-way ANOVA with post-hoc Tukey-Kramer Multiple-Comparison test).

The viability of encapsulated FP ICCs was assessed using 6-CFDA and PI staining. The viability prior to transplantation was 84±1%. Once transplanted into C57BL/6 mice viability was reduced to 80±1% at day 1 post-transplantation and cell death increased further especially at days 14 to 21 post-transplantation, with viability declining to a nadir of 20±4% at day 21 (Figures 2A&B). The low viabilities seen at days 14 and 21 post-transplantation correlated with severe PFO overgrowth (Table 1), which would be expected to block the passage of nutrients to the encapsulated cells.

#### Loss of FP ICC architecture by day 7 post-transplantation

**Figure 3:**
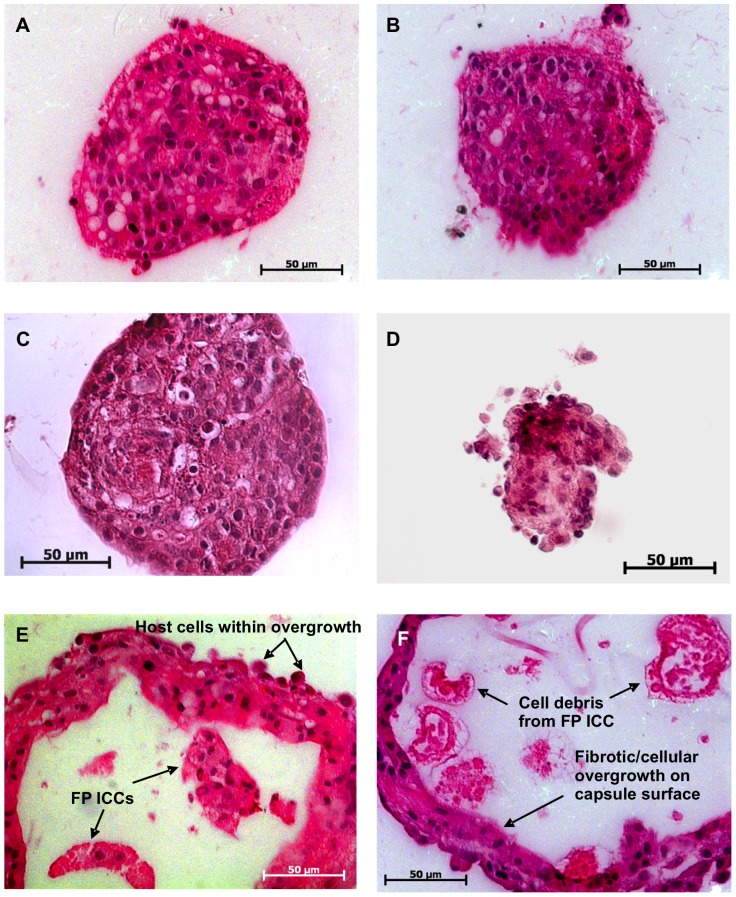
Histological analysis of microencapsulated FP ICCs. Microencapsulated FP ICCs at (A) pre-transplantation, (B) day 1, (C) day 3, (D) day 7, (E) day 14 and (F) day 21 post-transplantation. Morphology of encapsulated FP ICCs at days 1 and 3 was similar to pre-transplantation controls. Loss of FP ICC architecture and PFO was evident from days 7 to 21 indicating cell death associated with presence of cellular debris.

Paraffin sections of encapsulated FP ICCs were examined for their morphology with H&E staining to assess graft survival. Similar to the pre-transplantation controls (Figure 3A), encapsulated FP ICCs retrieved at days 1 and 3 post-transplantation (Figures 3B&C) were healthy and had intact islet structure with no host cells adherent to the surface of the recovered microcapsules. Smaller FP ICCs were observed from days 7 to 21 post-transplantation, with loss of islet architecture (Figures 3D–F). At day 21 post-transplantation cellular debris was observed without nuclear staining. Several layers of PFO were observed at days 14 to 21 post-transplantation (Figures 3E&F).

### Intragraft porcine transcript expression

**Figure 4:**
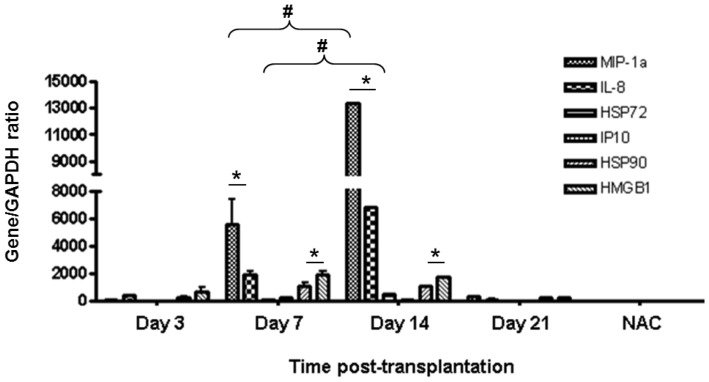
Intragraft porcine transcript expression in encapsulated FP ICCs. Increased intragraft transcript expression of porcine MIP-1α, IL-8, HSP90 and HMGB1 were seen within the first two weeks post-transplantation. The gene/GAPDH ratios were multiplied by 10,000 for clarity purposes. NAC (non-amplification control) represents the negative-RT sample analysed. Values are mean ± SEM (n = 4 mice for each time point); *p<0.05 for MIP-1α, IL-8, HSP90 and HMGB1 expression levels on days 7 and 14 when compared to days 3 and 21 post-transplantation (one-way ANOVA with post-hoc Tukey-Kramer Multiple-Comparison test); #p<0.05 for MIP-1α and IL-8 expression levels when compared between days 7 and 14 post-transplantation (Student's t-test).

Analysis of the cDNA synthesised from decapsulated FP ICCs recovered at various time points post-transplantation revealed an increase in the expression of pro-inflammatory factors macrophage inflammatory protein-1 alpha (MIP-1α) and interleukin-8 (IL-8) especially at days 7 and 14 compared to day 3 (Figure 4). A similar expression pattern was observed with the chaperone heat shock protein 90 (HSP90) and the high mobility group B1 (HMGB1) with levels peaking at days 7 and 14, respectively. Levels of porcine MIP-1α, IL-8, HSP90 and HMGB1 were very low in FP ICCs recovered 3 days post-transplantation, and returned to low values in those recovered 21 days post-transplantation. In contrast, levels of interferon gamma-induced protein 10 (IP10) and heat shock protein 72 (HSP72) remained low at all time points examined. The increased expression of the pro-inflammatory factors MIP-1α and IL-8 by FP ICC are consistent with the inflammatory response associated with mild to severe PFO and decrease in cell viability seen on days 7 and 14 post-transplantation respectively (Table 1 & Figure 2).

### Host immune cells and cytokines associated with PFO formation

#### Fibroblasts and macrophages are seen within the PFO

**Figure 5:**
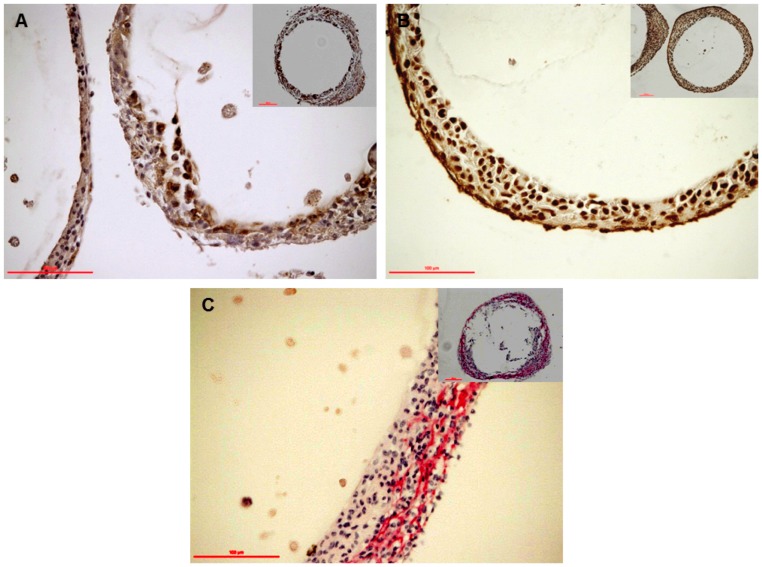
Immunohistological analysis of host immune cells associated with PFO. (A) Macrophages (F4/80+ cells) and (B) myofibroblasts (α-smooth muscle actin+ cells) were present in the PFO. (C) Positive picro-sirius red staining indicating the presence of collagen matrix. Micrographs are representative of grafts retrieved at day 21 post-transplantation.

H&E staining revealed the attachment of host cells and fibrosis on the surface of the capsules (Figure 3). We investigated the types of host cells within the PFO. Macrophages and fibroblasts appeared to be the dominant cell types that constituted the PFO from days 7 to 21 post-transplantation. This was shown by positive staining for F4/80 which is specific for macrophages (Figure 5A), and alpha-smooth muscle actin staining for myofibroblasts (Figure 5B). The presence of collagen was confirmed with picro-sirius red staining as shown in Figure 5C.

#### Intraperitoneal cytokines TNF-α, IL-4 and IL-5 are associated with PFO formation

**Figure 6:**
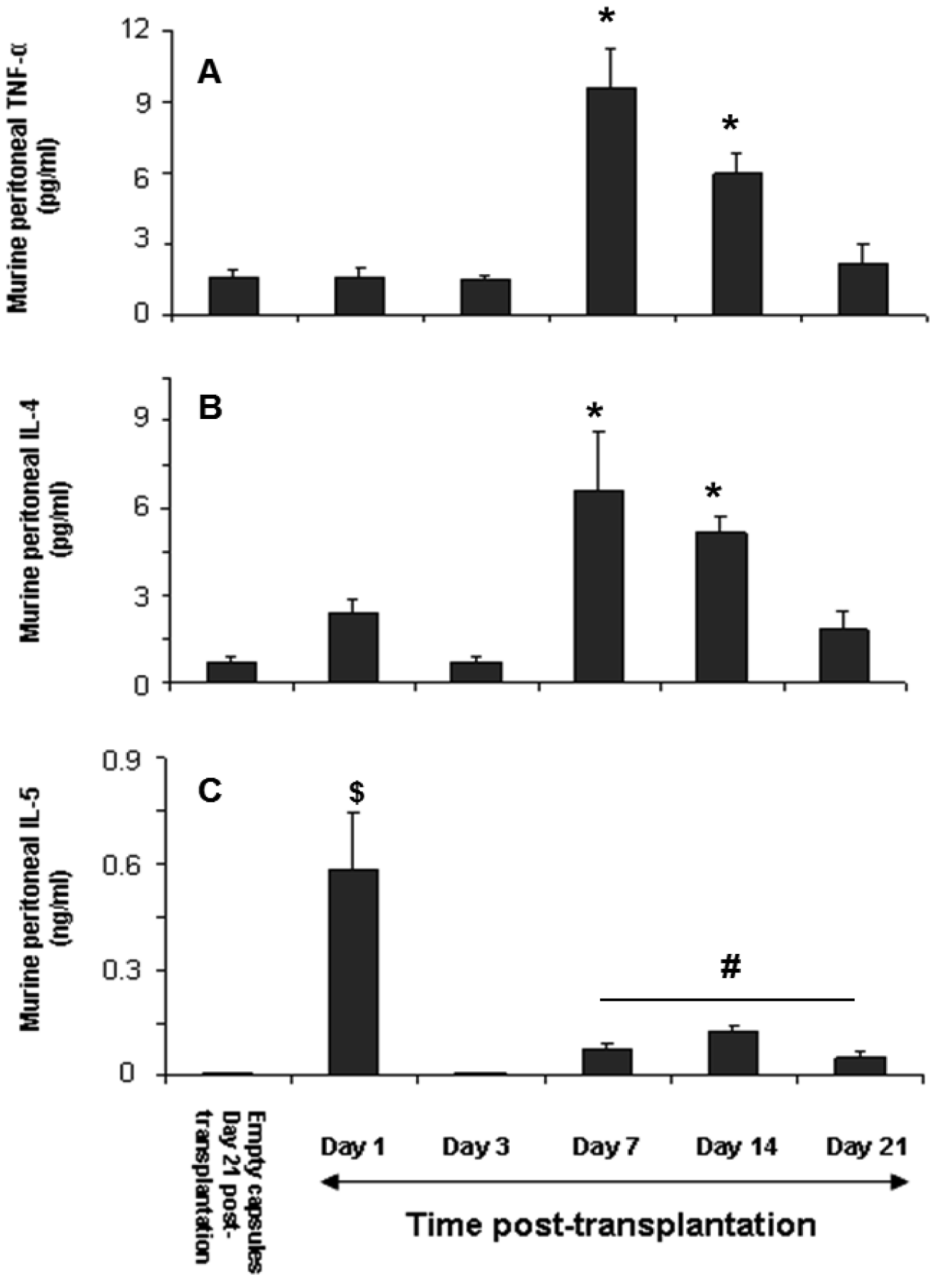
Determination of murine pro- and anti- inflammatory cytokines. Among the cytokines analysed, the only pro-inflammatory cytokine detected in the mouse peritoneal fluid was A) TNF-α and the anti-inflammatory cytokines detected were B) IL-4 and C) IL-5. Values are mean ± SEM (n = 4; for each time point); *p<0.05 for TNF-α and IL-4 levels on days 7 & 14 when compared to days 1, 3, 21 post-transplantation and empty capsules retrieved on day 21 post-transplantation; $p<0.05 for IL-5 levels on day 1 when compared to days 3, 7, 14, 21 post-transplantation and empty capsules retrieved on day 21 post-transplantation; #p<0.05 for IL-5 levels on days 7, 14 and 21 when compared to day 3 and empty capsules retrieved on day 21 post-transplantation (one-way ANOVA with post-hoc Tukey-Kramer Multiple-Comparison test).

As the fibrotic/cellular overgrowth was evident by day 7 post-transplantation, we explored which cytokines were associated with the PFO. The peritoneal fluid was collected from mice transplanted with empty microcapsules at day 21 post-transplantation and those transplanted with encapsulated FP ICCs at days 1, 3, 7, 14 and 21 respectively. The peritoneal fluid was subsequently concentrated and analysed for both pro- and anti-inflammatory cytokines. The only pro-inflammatory cytokine detected in the peritoneal fluid was TNF-α with levels peaking significantly at days 7 and 14 post-transplantation (Figure 6A) when mild to severe PFO was evident (Table 1). The levels of TNF-α decreased subsequently and reached basal levels at day 21 post-transplantation. A similar pattern was also seen with the multifunctional cytokine IL-4 with protein levels peaking at days 7 and 14 and subsequently reaching basal levels by day 21 (Figure 6B). However, other pro-inflammatory cytokines such as IL-1β, IL-2, IL-12 and IFN-γ were undetectable at all time points post-transplantation. On the other hand, levels of the anti-inflammatory cytokine IL-5 increased significantly one day after grafting, but declined thereafter to basal levels by day 21 post-transplantation (Figure 6C). The anti-inflammatory cytokine IL-10 was undetectable at all time points post-transplantation.

### Effect of the host xenogeneic immune response on graft function

#### Few insulin-positive cells were observed

**Figure 7:**
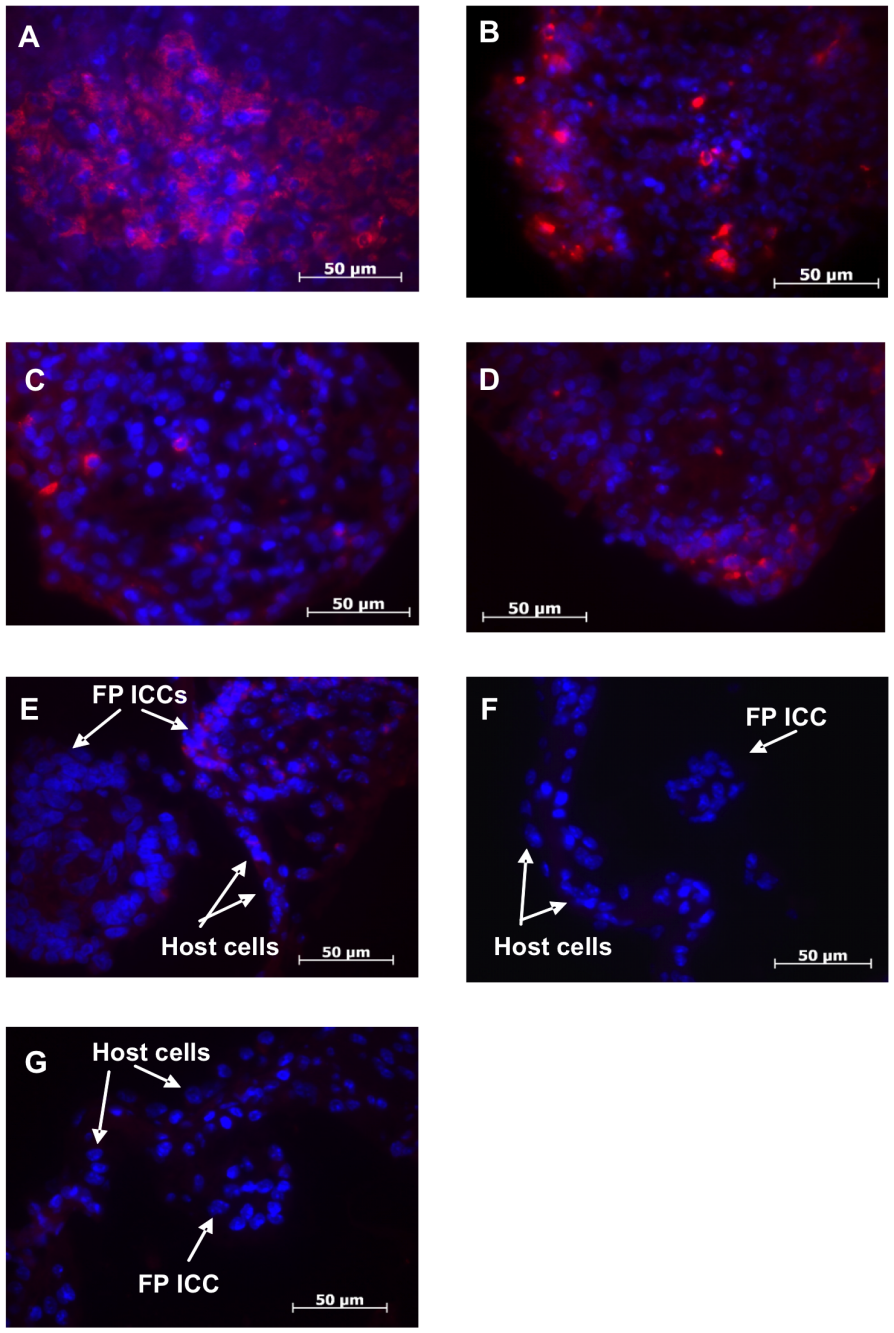
Immunofluorescent insulin staining of microencapsulated FP ICCs. (A) Insulin staining in adult pig pancreas (positive control), (B–G) microencapsulated FP ICCs at (B) pre-transplantation, (C) day 1, (D) day 3, (E) day 7, (F) day 14 and (G) day 21 post-transplantation. Insulin was stained with TRITC (in red) and nuclei stained with DAPI (in blue).

To investigate whether there were insulin-positive cells in the encapsulated FP ICCs, paraffin sections of the retrieved graft were stained for insulin. A small number of insulin-positive cells were identified in encapsulated FP ICC pre-transplantation, but the number of insulin-positive cells was less than the positive control, non-encapsulated adult pig pancreas (Figures 7A&B). Few insulin-positive cells were observed in FP ICCs at days 1 to 3 post-transplantation (Figures 7C&D), and this decreased between days 7 and 14 post-transplantation (Figures 7E&F), as PFO commenced. Insulin positive cells were absent in the small FP ICCs by day 21 post-transplantation which had a massive PFO (Figure 7G).

#### Insulin secretion and content of retrieved graft decreased post-transplantation

**Figure 8:**
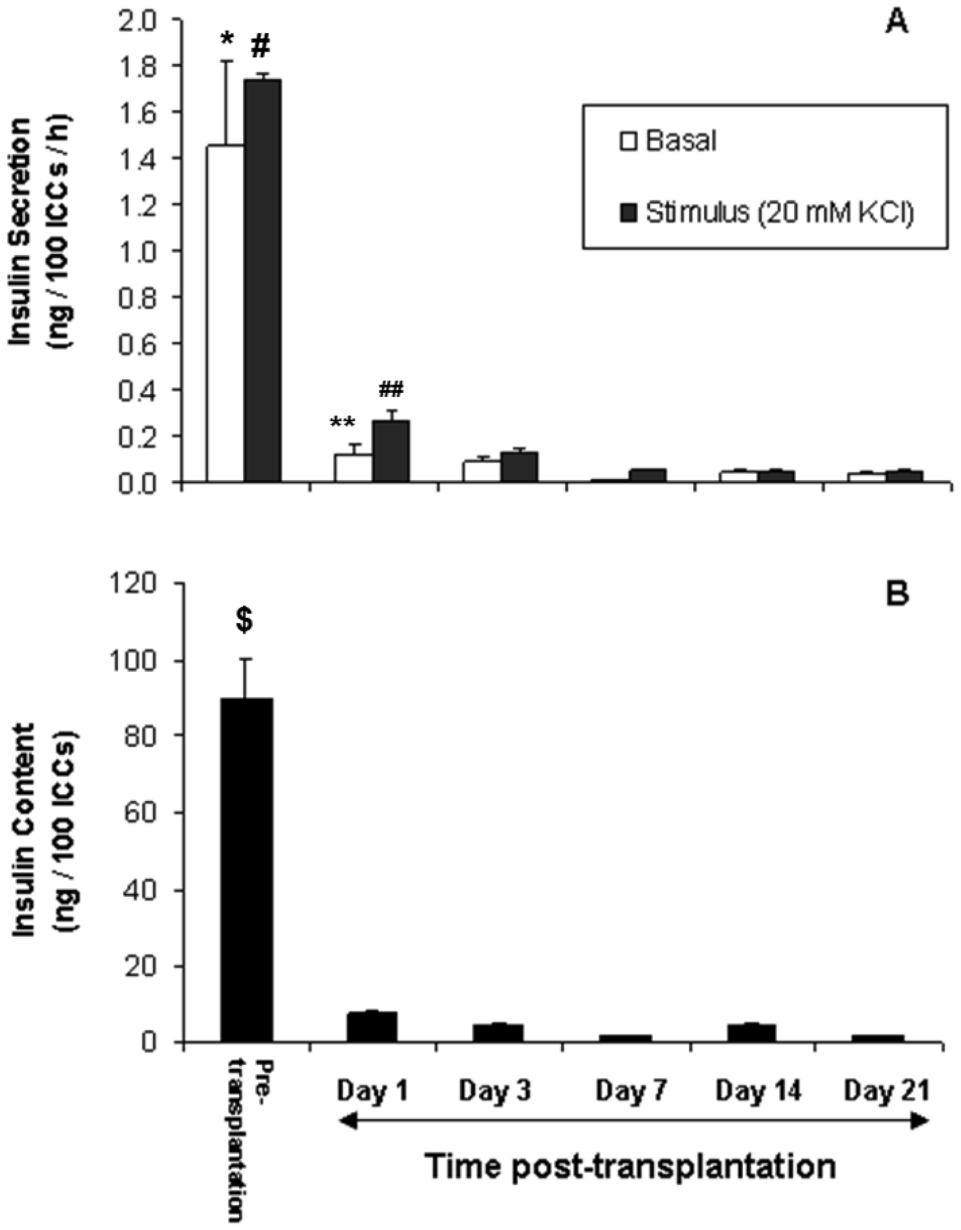
Insulin secretion from and insulin content of encapsulated FP ICCs. A) Recovered microencapsulated FP ICCs were stimulated ex vivo with a stimulus of 20 mM KCl in basal media (black bars) or with control basal media alone (white bars) to measure their insulin secretion. B) Ex vivo insulin content measurements on recovered microencapsulated FP ICCs after static stimulation. Values are mean ± SEM (n = 3 for pre-transplantation control and each time point post-transplantation); *,#p<0.05 for basal and stimulated insulin secretion levels of pre-transplantation controls when compared to all time points post-transplantation; **p<0.05 for basal insulin secretion level on day 1 compared to days 7, 14 and 21 post-transplantation; ##p<0.05 for stimulated insulin secretion level on day 1 compared to days 3, 7, 14 and 21 post-transplantation; $p<0.05 for insulin content level of pre-transplantation controls when compared to all time points post-transplantation (one-way ANOVA with post-hoc Tukey-Kramer Multiple-Comparison test).

As insulin staining showed the presence of few insulin-positive cells within the encapsulated FP ICCs, ability of the grafts to secrete insulin ex vivo was analysed. Encapsulated FP ICCs recovered from grafted mice were exposed to the insulinogenic stimulus 20 mM KCl and secreted insulin levels were measured. Encapsulated FP ICCs prior to transplantation secreted 1.7±0.02 ng/100 ICCs/h insulin after exposure to KCl and this level declined significantly to 0.28±0.04 ng/100 ICCs/h in FP ICCs recovered one day after transplantation (Figure 8A). Levels continued to decline thereafter reaching low levels by day 21 post-transplantation. The insulin content of FP ICCs followed a similar trend. Levels were 88±10 ng/100 ICCs prior to transplantation, declining to 8±0.1 ng/100 ICCs on day 1 and 1 ng/100 ICCs on day 21, respectively (Figure 8B).

#### Lack of graft function as evident by non-detectable porcine C-peptide levels

To determine if PFO might affect graft function, the level of porcine C-peptide in the host mouse sera was measured. This hormone was undetectable at all time points both before and after onset of PFO on day 7; suggesting that immaturity of the tissue [22], rather than blockage of nutrient supply was the reason. The controls, sera from mice transplanted with empty microcapsules, similarly had non-detectable levels of porcine C-peptide.

## Discussion

Previous studies with non-encapsulated FP ICCs transplanted into immunocompetent mouse recipients demonstrated heavy infiltration of CD4^+^ T cells and macrophages within day 5 post-transplantation and complete graft rejection within 8 to 14 days [23]–[25]. However, in our study encapsulated FP ICCs experienced no PFO by day 3 post-transplantation and only a slight fibrotic overgrowth by day 7 (Table 1). The extent of PFO correlated well with cell viability. This improved survival of FP ICCs may be attributed to the alginate microcapsules acting as a physical barrier to prevent direct contact of the FP ICCs with host immune cells. Although the alginate microcapsule improved graft survival, it can only offer a partial protection as the graft viability decreased with marked PFO which is evident even by day 14 post-transplantation. This is consistent with previous findings which showed a negative correlation between the PFO and cell viability with anti-porcine antibodies being detected in mouse sera within 5 to 14 days post-transplantation [14], [17]. The possibility that an immune response is not elicited against the alginate material itself can be ruled out as empty barium alginate microcapsules retrieved at 21 days post-transplantation were free of PFO consistent with our earlier studies [21], [26]. These findings suggest that xenoantigens and cytokines permeating through the porous alginate microcapsules lead to inflammation and the PFO. We have previously determined that the pore size of these alginate microcapsules would allow the trafficking of proteins up to ∼250 kDa in size [26]. The increased intragraft mRNA expression of porcine MIP-1α, IL-8, HMGB1 and HSP90 seen at days 7 and 14 post-transplantation suggest that small xenoantigens such as these could diffuse through the microcapsule pores and initiate an immune response which correlates with the appearance of PFO seen at day 7.

Macrophages are key players in chronic inflammation because of their ability to produce varied biologically active products and are known to be involved in immune responses against xenotransplanted microencapsulated islets [27], [28]. Siebers *et al.,* demonstrated that xenografting microencapsulated adult pig islets into the peritoneal cavity of rats resulted in the recognition of xenoantigens by the host macrophages with subsequent activation of them [27]. Similarly, in our study macrophages were seen within the PFO as part of the inflammatory reaction towards the xenografted encapsulated FP ICCs. This host xenogeneic immune response towards the encapsulated FP ICCs might be a delayed hypersensitivity response (DHR), which does not require T cells, but does involve macrophages and natural killer cells [29], [30]. The increase in the intragraft mRNA expression of MIP-1α at days 7 and 14 is consistent with a previous finding [31], and the role of this chemokine in recruiting macrophages to initiate the inflammatory process resulting in PFO seen on days 7 and 14, respectively. Previously, it has been shown that heat shock proteins (HSP) which are released upon cell death can activate macrophages as part of the innate immune response and stimulate them to secrete cytokines [32], [33]. In our study, although the protein levels were not measured, we found increased intragraft mRNA expression of HSP90 which correlated with a significant decrease in cell viability and appearance of PFO by day 7. Increased HSP90 expression seen at day 7 might have activated the peritoneal macrophages by an innate immune response and led to cytokine production. This possibility is further supported by the murine TNF-α protein levels measured in the peritoneal fluid with levels peaking at day 7 post-transplantation. It is known that the effects of activated macrophages are mediated by the production of pro-inflammatory cytokines such as TNF-α and reduced levels in serum has been correlated with macrophage depletion [34], [35]. In our study the increase in murine TNF-α levels seen at day 7 post-transplantation correlates with the onset of PFO and this is consistent with activated macrophages being an important source of this pro-inflammatory cytokine.

The cytokines, other than TNF-α which are detectable in the mouse peritoneal fluid and are involved in the xenogenic immune response leading to PFO, were IL-4 and IL-5. This observation is similar to previous studies where predominant changes in the expression of anti-inflammatory cytokines have been observed from days 4 to 8 post-transplantation [36]–[38]. It is known that activated immune cells release various cytokines such as IL-4, IL-5, IL-6, IL-10 and IL-13 and an immune response is elicited by increased levels of IL-4 [39]. In our study we found that the IL-5 and IL-4 levels peaked at days 1 and 7 respectively post-transplantation and their levels decreased with time thereafter. This is similar to a previous study where enhanced expression of IL-4 and IL-5 transcripts were seen within the first few days postransplantation (days 4 to 8) in pig proislet xenografts [40]. Interestingly, the anti-inflammatory cytokine IL-10, a potent suppressor of macrophage function and inhibitor of pro-inflammatory cytokine synthesis, was undetectable at any time post-transplantation. This might explain the presence of macrophages and the proinflammatory cytokine TNF-α leading to PFO seen at days 7 to 21 post-transplantation. Our findings are consistent with a previous study [41] where marked expression of IL-5 and TNF-α was observed but not IFN-γ in the pig pro-islet xenografts within the first few days following transplantation. Further, TNF-α is also involved in the chronic inflammatory process inducing fibroblast proliferation thereby producing extracellular matrix proteins such as collagen, proteoglycans and glycoproteins [42]. Indeed, we found that the PFO associated with chronic inflammation stained strongly for α-smooth muscle actin, a marker of activated myofibroblasts and collagen. This correlated well with the increase in intraperitoneal TNF-α on day 7 suggesting the possibility of TNF-α induced proliferation of active pericapsular fibroblasts leading to PFO. Based on the above evidence, it can be postulated that the macrophages are direct effectors of the xenorejection of encapsulated FP ICC leading to PFO mediated by a combination of both pro-and anti-inflammatory cytokines.

The PFO associated with an inflammatory response is likely to hinder graft function from day 7 post transplantation as passage of nutrients becomes impaired. However, immaturity of the tissue appears to be the key factor as we were unable to detect porcine C-peptide at any stage during the 3 weeks post transplantation. We have previously demonstrated that it took at least a month for encapsulated FP ICCs to fully differentiate into insulin producing cells and normalise blood glucose levels when transplanted into immunocompromised diabetic recipients [12]. Moreover, when fetal pig pancreatic tissue was grafted into non-diabetic immunodeficient mice, porcine C-peptide was detectable in mouse serum only after 3 weeks [43]. The low levels of insulin secretion from and graft content of *ex vivo* encapsulated ICCs at days 1 and 3 post transplantation, as compared to levels obtained pre-transplantation, can best be explained as a response to a recent change in environmental conditions. Viability of the grafts was high at this time and pro-inflammatory cytokines were not detectable in mouse peritoneal fluid. Even if they had, fetal pig β cells [44], as compared to adult β cells [45], [46], have been shown to be resistant to the adverse effect of these cytokines.

In conclusion, our findings have further characterised the mechanism of xenogeneic immune response in terms of the intraperitoneal cytokines and immune cells involved in the rejection of microencapsulated FP ICCs. Encapsulated FP ICC xenograft rejection was associated with enhanced intragraft transcript expression of porcine antigens MIP-1α, IL-8, HMGB1 and HSP90 seen on days 7 to 14 post-transplantation. This pattern was consistent with the recruitment of macrophages and infiltration of activated myofibroblasts leading to PFO and loss of viability of the graft. This study also demonstrates that microencapsulation with barium alginate beads is not sufficient by itself to prevent the xenograft rejection of FP ICCs transplanted into the peritoneal cavity of immunocompetent mice. Strategies such as temporary macrophage depletion by clodronate and inhibition of co-stimulatory pathways by anti-LFA-1/anti-CD154 antibodies have resulted in prolonged survival of encapsulated porcine tissue [18], [47]. Thus, future research exploring the combination of microencapsulation and immunosuppressive strategies to overcome xenograft rejection will advance the possibility of using encapsulated FP ICCs as a treatment for insulin-dependent diabetes.

## Supporting Information

Figure S1 **Microencapsulation of FP ICCs.** The FP ICCs were encapsulated in a highly purified 2.2% alginate solution using an air-driven droplet generator. The picture represents the homogeneity of the microcapsules with an average diameter of 244.9±13.9 µm.
(TIF)

Table S1 **TaqMan probe and primer sequences used to detect procine mRNA expression by RT-PCR.**
(DOC)
