# Supplementary figures and images for: Characterisation of the Xenogeneic Immune Response to Microencapsulated Fetal Pig Islet-Like Cell Clusters Transplanted into Immunocompetent C57BL/6 Mice
Source: PLoS One. 2013 Mar 15;8(3):e59120. doi: 10.1371/journal.pone.0059120 (PMC3598741; doi:10.1371/journal.pone.0059120)

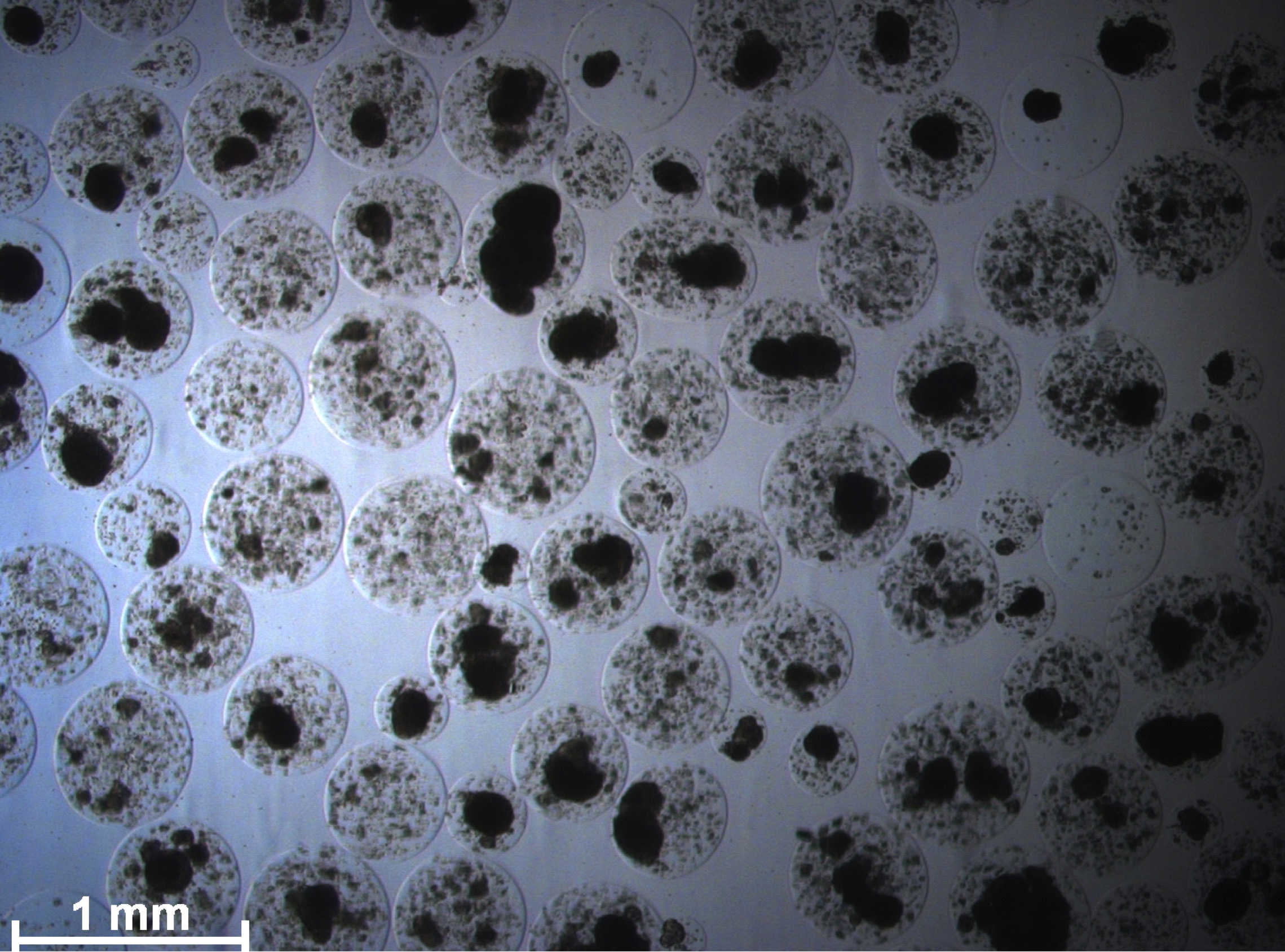

Supplement: Figure S1 — Microencapsulation of FP ICCs. The FP ICCs were encapsulated in a highly purified 2.2% alginate solution using an air-driven droplet generator. The picture represents the homogeneity of the microcapsules with an average diameter of 244.9±13.9 µm. (TIF) [file pone.0059120.s001.tif]
