# Supplementary material for: Characterisation of the Xenogeneic Immune Response to Microencapsulated Fetal Pig Islet-Like Cell Clusters Transplanted into Immunocompetent C57BL/6 Mice
Source: PLoS One. 2013 Mar 15;8(3):e59120. doi: 10.1371/journal.pone.0059120 (PMC3598741; doi:10.1371/journal.pone.0059120)
Supplement: Table S1 — TaqMan probe and primer sequences used to detect procine mRNA expression by RT-PCR. (DOC) [file pone.0059120.s002.doc]

Table S1. TaqMan probe and primer sequences used to detect porcine mRNA expression by RT-PCR

| **Gene** | **TaqMan forward primer sequence 5’ to 3’** | **TaqMan reverse primer sequence 5’ to 3’** | **TaqMan probe sequence 5’ to 3’** |
| --- | --- | --- | --- |
| MIP-1α | AGCGCTCTCAGCACCAATG | TTCCGCACGGTGTATGTGA | CAGACCCTCCCACCTCCTGCTGC |
| IL-8 | GGGTGGAAAGGTGTGGAATG | GGCTGCAGTTCTGGCAAGAG | TGCACTGGCATCGAAGTTCTGCACTT |
| IP-10 | TTGAAATGATTCCTGCAAGTCAA | TTTCTCCCCATTCTTTTTCATTG | CTTGCCCACATGTTGAGATCATTGCC |
| HMGB1 | AAGGAGAGCATCCTGGCCTATC | TCTGCAGCGGTGTTATTCCA | TGGTGATGTTGCAAAGAAACTGGGAGAGAT |
| HSP72 | CCAGATTGAGGTGACCTTCGA | TGCCTGTGCTCCTGTCAGT | TGTCACACTCAGGATGCCATTGGCA |
| HSP90 | GGCCGGTGCCGATATCTC | AGCGACCAGGTACGCAGAGTA | AAGCCGACACCGAACTGGCCAA |
